# Characterization of Oral Melanocytic Nevi in Sun‐Exposed and Sun‐Protected Regions

**DOI:** 10.1111/odi.15404

**Published:** 2025-06-12

**Authors:** Thalita Soares Tavares, Adriana Aparecida Silva da Costa, Marina Gonçalves Diniz, Daniela Pereira Meirelles, Sandra Beatriz Chaves Tarquínio, Ana Carolina Uchoa Vasconcelos, Aline Cristina Batista Rodrigues Johann, Ricardo Santiago Gomez, Maria Cássia Ferreira de Aguiar, Patrícia Carlos Caldeira

**Affiliations:** ^1^ Department of Oral Pathology and Surgery, School of Dentistry Universidade Federal de Minas Gerais Belo Horizonte Minas Gerais Brazil; ^2^ Department of Pathology, Biological Science Institute Universidade Federal de Minas Gerais Belo Horizonte Minas Gerais Brazil; ^3^ Department of Semiology and Clinics, School of Dentistry Universidade Federal de Pelotas Pelotas Brazil; ^4^ Department of Dentistry, School of Life Sciences Pontifícia Universidade Católica Do Paraná Curitiba Brazil

**Keywords:** MiTF protein, mouth, oral nevus, TRP2 protein

## Abstract

**Objective:**

Oral melanocytic nevi (OMN) are histologically similar to skin nevi; however, they are much rarer and not always related to sun exposure. This study aimed to characterize the histopathological, immunohistochemical, and molecular features of OMN in sun‐exposed (vermilion lip) and sun‐protected (intraoral) regions.

**Method:**

Cross‐sectional study on 14 intraoral and 20 vermilion lip OMN. Hematoxylin–eosin, immunohistochemistry for TRP2 and MiTF, and RT‐qPCR for TRP2 and MiTF were used to assess histopathology, protein, and mRNA expression, respectively. Statistical analysis was performed.

**Results:**

Intramucous and blue nevi are the most frequent histological subtypes. Intraoral lesions predominantly affect Brown or Black individuals (64.3%), presenting mostly as macules. Vermilion lip nevi affect White individuals (75%), with elevated lesions (30%). Histologically, intraoral nevi show asymmetric, lack theques formation and floret cells, and present no mitotic activity or pleomorphism, while vermilion nevi display pleomorphism, symmetry, theques formation, floret cells, and solar elastosis. TRP2 and MiTF protein and mRNA expression were upregulated in both groups (*p* < 0.05), with no differences between them (*p* > 0.05).

**Conclusions:**

Intraoral and vermilion lip nevi have distinct histopathological features, but similar TRP2 and MiTF protein and mRNA expression. This characterization may aid the histopathological diagnosis of OMN and distinction from other oral pigmentations.

## 1 Introduction

Oral melanocytic nevi (OMNs) are rare benign pigmented neoplasms generally arising as a single macule, papule, or nodule in young adults (Tavares et al. 2021). The etiology of melanocytic nevi is multifactorial and heterogeneous, with multiple molecular events contributing to their development (Roh et al. 2015). Cutaneous nevi frequently harbor a BRAF^V600E^ mutation which lacks an ultraviolet B (UVB) signature (Takata and Saida 2006), even though these lesions usually arise in sun‐exposed regions. Accordingly, OMNs have been reported to harbor BRAF^V600E^ mutations despite not being associated with exposure to UVB radiation (Resende et al. 2021). The histological and molecular differences between OMNs in sun‐exposed (lip vermilion) and sun‐protected (intraoral) sites have been seldom reported. A better understanding of how anatomical location influences the biological behavior of OMNs could provide valuable insights into their differential diagnosis and clinical management.

Of interest, nevus‐to‐melanoma transformation has been discussed, and in 2018 the WHO classified nine melanoma evolutionary pathways based on genetic alterations, precursor lesions, and chronic sun damage (Elder et al. 2020). Previous studies have demonstrated a distinct mutational spectrum between melanomas arising in sun‐shielded versus sun‐exposed areas (Furney et al. 2013). Cutaneous melanomas arising from pre‐existing nevus often involve BRAF mutations, whereas de novo melanomas more frequently exhibit UV‐induced mutations like TP53 (Shreberk‐Hassidim et al. 2023). Notably, no precursor benign lesion has been established for mucosal melanoma yet, and this subject is still a matter of debate (Elder et al. 2020; Meleti et al. 2007; Queiroz et al. 2025). After acquiring BRAF^V600E^ mutation, OMNs enter senescence; thus, its eventual progression into mucosal melanoma would need additional genetic alterations (Vredeveld et al. 2012).

Melanocyte‐specific proteins, such as Tyrosinase Related Protein‐2, also known as Dopacrome Tautomerase (TRP2/DT), and the Microphthalmia Transcription Factor (MiTF), are crucial regulators of melanogenesis (Filimon and Negroiu 2009; Lu et al. 2010), and both have been identified in skin benign and malignant pigmented lesions (Granter et al. 2001; Itakura et al. 2008; King et al. 2001; Miettinen et al. 2001). Given their central role in the melanogenesis pathway, we hypothesize that aberrant expression or dysfunction of these proteins may be associated with oral melanocytic lesions. Furthermore, we propose that OMNs from sun‐shield (intraoral) and those from sun‐exposed regions (vermilion of the lip) may exhibit differential expression of these melanocyte‐specific proteins, reflecting distinct etiological pathways. As far as we know, this is the first study to examine TRP2/DT and MiTF expression in OMNs.

Therefore, the present study aims to describe the histopathological characteristics and to investigate the expression of TRP2/DT and MiTF in OMNs, comparing lesions from sun‐exposed areas (vermilion of the lip) with those from sun‐protected areas (intraoral) using immunohistochemistry and RT‐qPCR.

## 2 Material and Methods

### 2.1 Study Design, Sampling, and Ethical Aspects

This cross‐sectional study was performed with a convenience sample of thirty‐four formalin‐fixed, paraffin‐embedded (FFPE) intraoral (*n* = 14) and vermilion lip (*n* = 20) acquired nevus tissue samples. The acquired nature of all nevus was confirmed based on the description on the clinical cards. All material was obtained from the archives of three Oral Pathology Services of Schools of Dentistry in Brazil: *Universidade Federal de Minas Gerais*, *Universidade Federal de Pelotas*, and *Pontifícia Universidade Católica do Paraná*.

The inclusion criteria were cases with a histopathological diagnosis of intraoral and vermilion lip‐acquired melanocytic nevus (junctional, compound, and intramucosal) and blue nevus. Cases with another diagnosis of nevus, such as halo nevus, Spitz nevus, dysplastic nevi, and congenital melanocytic nevus, were excluded. No clinical or demographic characteristic was set as inclusion or exclusion criteria. Sex, age, race, lesion site, clinical appearance, color, and size were retrieved from patient records. All the hematoxylin and eosin (H&E)–stained sections were reviewed by one oral pathologist (P.C.C.) to confirm the diagnosis and histological subtype, and accomplishment with inclusion and exclusion criteria. Cases with doubtful histopathological features were excluded.

This study was performed in accordance with the Declaration of Helsinki and was approved by the Committee of Ethics in Research of *Universidade Federal de Minas Gerais* (certificate number 57392522.6.0000.5149). All patient data were anonymized to ensure confidentiality and compliance with ethical standards.

### 2.2 Histopathological Evaluation

Two examiners (T.S.T. and P.C.C.) simultaneously evaluated the H&E‐stained sections using a binocular light microscope. The entire slide was screened and analyzed at both 100× and 400× magnifications. The histopathological features assessed (Ahn et al. 2016) were the lateral circumscription, symmetry, stromal reaction (inflammation, fibrosis, and solar elastosis), theques formation, floret cell presence, and mitotic activity. Additionally, alterations in the rete ridges (classified as absent, elongated, or elongated with bridge formation), the degree of pleomorphism and melanin deposition (categorized as absent, mild, moderate, or intense), and the morphology of the cells (round only, fusiform only, most round with few fusiform, or most fusiform with few round) were recorded.

### 2.3 RT‐qPCR

Total RNA was extracted from intraoral nevus (*n* = 14), vermilion lip nevus (*n* = 20), and oral mucosa (*n* = 52) FFPE samples (30 to 40 sections of 5 μM thickness) using the ReliaPrep FFPE Total RNA Miniprep System (Promega Biotectologia do Brasil LTDA, cat#Z1002) according to the manufacturer's instructions. RNA quantity was assessed using a NanoDrop 2000 spectrophotometer (Thermo Fisher Scientific, MA, USA), and all RNA samples were stored at −80°C. SuperScript VILO cDNA Synthesis Kit (Invitrogen, Massachusetts, USA, cat#11754050) was used for synthesizing cDNA from 1500 ng of RNA, according to manufacturer's guidelines. The qPCR reactions were conducted with the PowerUp SYBR Green Master Mix (Applied Biosystems, Massachusetts, USA, cat#A25742) with the following primers: TRP2: 5′‐CGAAACCAGGATGACCGTGA‐3′ (F) and 5′‐TAGCCGGCAAAGTTTCCTGT‐3′ (R); MiTF: 5′‐TCACCATCAGCAACTCCTGTC‐3′ (F) and 5′‐TGTGGGAAAAATACACGCTGTGAG‐3′ (R). The reactions were carried out in duplicate on a StepOnePlus Real‐Time PCR System thermocycler (Thermo Fisher Scientific). mRNA expressions were normalized using GAPDH as the reference gene and values were obtained with the formula 2^−(∆Ct)^.

### 2.4 Immunohistochemistry (IHC)

IHC was performed following standard procedures using the monoclonal antibodies TRP2/DCT (ab221144; dilution 1:2000; Abcam, REF EPR21986) and MiTF (Clone: D5, dilution 1:150; Invitrogen, REF MA5‐14154). Four‐micron‐thick sections were cut and mounted on glass slides for IHC. The sections were dewaxed in xylene and rehydrated in decreasing concentrations of alcohol. We incubated the tissues in Tris/EDTA buffer (pH 9.0) for antigen retrieval in a pressure cooker at 96°C for 30 min. Endogenous peroxidase was blocked using EnVision FLEX Peroxidase‐Blocking Reagent (15 min incubation at room temperature). Afterward, monoclonal antibodies directed against TRP2/DCT and MiTF were incubated at room temperature for 60 min. For TRP2 and MiTF, the slides were additionally incubated with EnVision FLEX+ Rabbit LINKER and Mouse LINKER, respectively (15 min at room temperature) to enhance the signal. Detection was undertaken with a ready‐to‐use polymer (Envision FLEX/HRP). Next, the reaction was revealed with PermaRed/HRP (Diagnostic BioSystems, code K075) and counterstained with hematoxylin. Negative controls were obtained by the omission of primary antibodies, while sections of oral melanotic macule served as positive controls.

### 2.5 Evaluation of Immunohistochemistry

IHC‐stained sections were evaluated simultaneously by two examiners (T.S.T and P.C.C.) using a binocular light microscope. Nuclear MiTF and cytoplasmic TRP2/DCT expression were evaluated semi‐quantitatively based on the percentage of positively stained cells in the entire lesion and coded as 0: no staining; 1: < 25%; 2: 25%–75%; and 3: > 75%.

### 2.6 Statistical Analysis

Histopathological and immunohistochemical results were submitted to descriptive statistics, using the SPSS version 26.0 software (SPSS Inc., Armonk, NY, USA). Statistical analysis of RT‐qPCR was performed using GraphPad Prism software, version 8.0 for MacOS X (San Diego, CA, USA). The data were analyzed for homogeneity using the Shapiro–Wilk test. To compare mRNA expression between groups, the Kruskal‐Wallis test was employed, followed by Dunn's post hoc test for multiple comparisons (*p*‐values adjusted to account for multiple comparisons). Correlation between mRNA expression levels was evaluated using Pearson's correlation coefficient for parametric data or Spearman's correlation coefficient for non‐parametric data. For all data, a 95% confidence interval and a significance level of 0.05 were used.

## 3 Results

Table S1 summarizes the clinical and demographic data for the two groups, considering the histological subtypes. Intraoral nevi arose mainly at the hard palate (*n* = 5, 35.7%), while the lower lip was more frequent for the vermilion lip lesions (*n* = 12, 60.0%). Intraoral and vermilion lesions were similar at affecting mostly females (~70%), with a wide age range (11 to 50 years old, ~75.0%), and presenting as small lesions (0‐5 mm, ~50.0%). However, some differences were observed: Intraoral lesions affected mostly Brown or Black people (*n* = 9, 64.3%), and were mostly macular lesions (*n* = 9, 64.3%), while lip vermilion affected most White people (*n* = 15, 75.0%) and arose as elevated lesions (*n* = 6, 30.0%).

**FIGURE 1:**
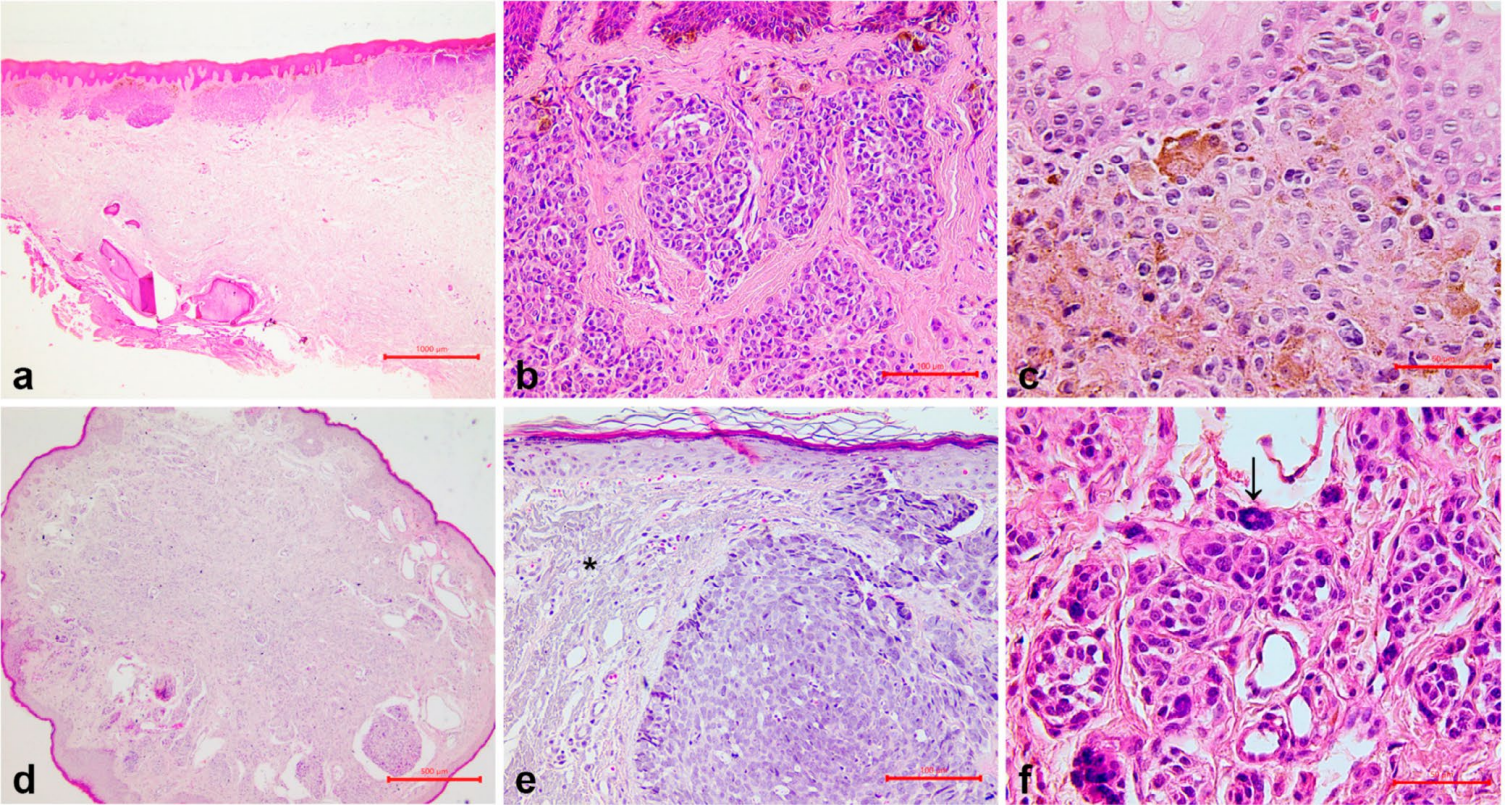
Histological features of oral nevi. (a) Intraoral compound melanocytic nevus, displaying lateral circumscription, melanin deposition in superficial nevus cells (close to epithelium), asymmetry, and absence of theques formation. H&E, 20×. (b) Intraoral compound melanocytic nevus showing round cells grouped in theques, fibrosis of the connective tissue, no inflammation, lack of mitosis, and focal melanin deposition. H&E 200×. (c) Intraoral intramucosal melanocytic nevus, with round cells containing melanin, lacking theques formation, mitosis, and pleomorphism. H&E, 400×. (d) Vermilion lip intramucosal melanocytic nevus demonstrating nodular growth, lateral circumscription, and symmetry. H&E, 40×. (e) Vermilion lip intramucosal melanocytic nevus with round and fusiform cells organized in nests, mild pleomorphism, and solar elastosis (asterisk) in the connective tissue. H&E, 200×. (f) Vermilion lip compound melanocytic nevus, with round cells, theques formation, and mild pleomorphism, including floret cells (arrow). H&E, 400×.

Table S2 and Figure 1 show the results of the histological examination. Lesions in both groups predominantly exhibited fibrosis (~65.0%), mild melanin deposition (~50.0%), absence of lateral circumscription (55.0% to 64.0%), no inflammatory response (75.0% to 92.0%), unchanged rete ridges (> 85.0%), and lack of mitotic activity (> 95.0%). Despite these similarities, significant differences were observed between the groups: Intraoral lesions were mostly asymmetrical (*n* = 8, 57.1%), with no theques formation (*n* = 11, 78.6%), absence of floret cells (*n* = 13, 92.9%), and absence of pleomorphism (*n* = 9, 64.3%). The cells in intraoral lesions were either round (*n* = 7, 50.0%) or fusiform (*n* = 6, 42.9%). In contrast, the vermilion lip group was predominantly symmetrical (*n* = 17, 85.0%), with the presence of solar elastosis (*n* = 14, 70.0%), presence of theques formation (*n* = 16, 80.0%), presence of floret cells (*n* = 13, 65.0%), and presence of pleomorphism, which was mild (40.0%) or moderate (35.0%). Cells in the vermilion lip group were mainly round (*n* = 18, 90.0%).

**FIGURE 2:**
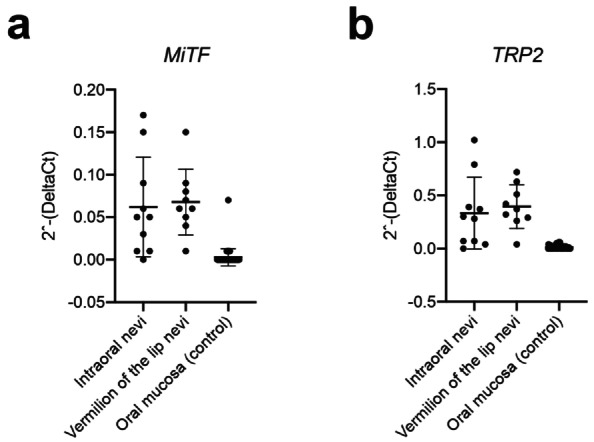
MiTF (a) and TRP2 (b) mRNA expression. mRNA expression was calculated with the 2^−(∆Ct)^ formula, using GAPDH as the reference gene. Horizontal bars represent the mean (SD). Kruskal–Wallis with post hoc Dunn's multiple comparison tests were used.

Overall, TRP2 and MiTF expression were similar across groups, pointing that UV exposure does not appear to influence the expression of these markers in OMNs. RT‐qPCR analysis showed a significant upregulation (*p* < 0.0001 for both genes) of MiTF (Figure 2a; intraoral mean 0.063, SD 0.055; vermilion of the lip mean 0.069, SD 0.036) and TRP2 (Figure 2b; intraoral mean 0.331, SD 0.319; vermilion of the lip mean 0.395, SD 0.192) in intraoral and vermilion of the lip OMN compared to the oral mucosa (MiTF mean 0.004, SD 0.010; TRP2 mean 0.013, SD 0.017). However, no significant difference was observed in the expression levels of MiTF and TRP2 (*p* > 0.9999 for both genes) between intraoral and the vermilion lip sites (Figure 2a,b). A positive correlation was observed between TRP2 and MiTF expression in vermilion of the lip nevi (*r* = 0.74; *p* = 0.02) and in intraoral nevi (*r* = 0.61; *p* = 0.059).

**FIGURE 3:**
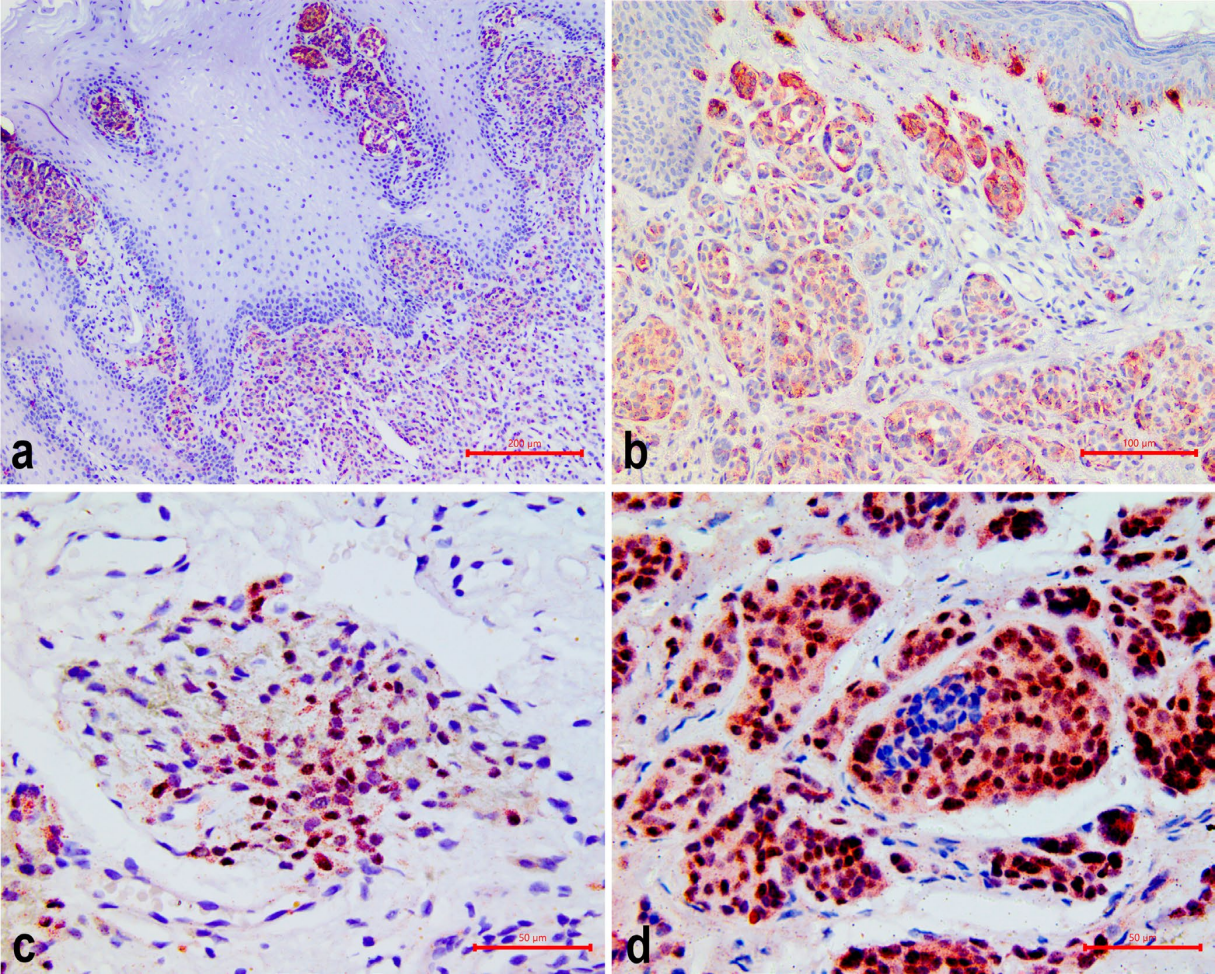
Immunohistochemical expression of melanocyte‐specific markers. (a) TRP2 cytoplasmic immunostaining in a case of intraoral nevus, classified with score 2 (25%–75% of cells stained), showing higher positivity in superficial melanocytes (near epithelium). (b) TRP2 cytoplasmic immunostaining in a case of vermilion lip nevus, also classified with score 2 and demonstrating increased expression in superficial cells. (c) MiTF immunostaining in an intraoral nevus, highlighting a nuclear expression. (d) MiTF immunostaining in a vermilion lip nevus, showing the nuclear staining. HRP‐Permanent Red staining; magnifications: (A) ×100, (B) ×200, (C) ×400, (D) ×400.

The immunoexpression of TRP2 (Table 1) was observed in both intraoral and vermilion lip lesions, typically with a score 2 (25%–75% of positive cells). Intraoral lesions showed a slightly higher occurrence of this score (*n* = 8, 57.1%) compared to vermilion lesions (*n* = 8, 40.0%). Staining was less pronounced in deep regions of the slides of both groups (Figure 3a,b). Similarly, MiTF immunoexpression (Table 1) was observed in both groups, with a score 2 being more common. A higher proportion of vermilion lesions presented a score 2 for MiTF expression (*n* = 10, 50.0%) than intraoral lesions (*n* = 5, 35.7%). The staining was lower in the deep regions of intraoral nevi (Figure 3c,d).

**TABLE 1:** Immunohistochemical expression of MiTF and TRP2 in intraoral and vermilion lip nevi.

	Intraoral (*n* = 14)				Vermilion of the lip (*n* = 20)			
	Compound (*n* = 3)	Intramucosal (*n* = 5)	Blue (*n* = 6)	All (*n* = 14)	Compound (*n* = 6)	Intramucosal (*n* = 13)	Blue (*n* = 1)	All (*n* = 20)
TRP2								
% of positive cells								
0%	0	0	1	1 (7.1%)	0	0	0	0 (0.0%)
< 25%	0	0	2	2 (14.3%)	3	2	0	5 (25.0%)
25% to 75%	3	3	2	8 (57.1%)	3	5	0	8 (40.0%)
> 75%	0	2	1	3 (21.4%)	0	6	1	7 (35.0%)
MiTF								
% of positive cells								
0%	0	0	1	1 (7.1%)	0	0	0	0 (0.0%)
< 25%	0	0	4	4 (28.6%)	1	1	0	2 (10.0%)
25% to 75%	2	3	0	5 (35.7%)	3	6	1	10 (50.0%)
> 75%	1	2	1	4 (28.6%)	2	6	0	8 (40.0%)

## 4 Discussion

OMNs are uncommon congenital or acquired benign melanocytic tumors, histologically similar to their cutaneous counterparts (Hatch 2005). Consistent with previous studies, our findings showed that intramucosal nevi were the most common subtypes of acquired melanocytic nevi at both intraoral and vermilion lip sites (Ferreira et al. 2015; Meleti et al. 2007; Tavares et al. 2021). However, in contrast to the literature, we observed that intraoral nevi were more prevalent in individuals with darker skin tones, typically presenting as flat lesions, while vermilion lip lesions were more common in white individuals and appeared as elevated lesions. These observations suggest possible anatomical or environmental factors influencing the clinical presentation of OMNs.

In a retrospective review of 100 OMNs, 82 were in the oral cavity and 18 in the vermilion lip (Ferreira et al. 2015). Among these, two junctional nevi exhibited atypical features, including poor circumscription and large melanocytic nests near the rete tips, replacing the basal cell layer and resulting in a lentiginous appearance. These cases were subsequently reclassified as dysplastic nevi with mild atypia (Ferreira et al. 2015). Similarly, a review of nevi from special sites, including UVB‐protected regions such as the ear, ocular conjunctiva, genitalia, acral skin, scalp, breast, and other sites revealed atypical histopathological features despite their benign nature. These include asymmetry, irregular nesting patterns, pagetoid spread melanocytes, cytologic atypia, architectural disorders, large nests at the rete tips, and inflammatory infiltrates (Ahn et al. 2016). Consistent with this, our findings from intraoral nevi showed asymmetry, lack of lateral circumscription, and absence of theques formation, though they did not exhibit mitotic activity, pleomorphism, or inflammatory response. Vermilion lip lesions, while exhibiting more pleomorphism, were largely symmetrical, with theques formation, and showed solar elastosis. Multinucleated giant cells with hyperchromatic nuclei arranged in a wreath‐like pattern (floret cells) were observed in most vermilion lip cases and occasionally in intraoral nevi. To our knowledge, floret cells have not been previously described in these lesions and may represent a reactive or degenerative phenomenon rather than an indicator of malignancy. Further studies are needed to clarify their significance in these lesions.

The presence of atypical features observed in nevi from uncommon sites like the oral mucosa can lead to over‐diagnosis of melanoma, resulting in unnecessary treatments. In this regard, the absence of mitotic figures and lack of significant cytologic atypia in intraoral nevi, along with the symmetry observed in vermilion lip nevi, can help distinguish OMNs from malignant melanocytic lesions. Immunohistochemistry can help in doubtful cases, though its results come from studies of lesions from sites other than oral mucosa: HMB‐45 exhibits stratified expression in benign nevi, with loss of staining in deeper dermal components, whereas melanomas typically show diffuse expression. Tyrosinase is highly expressed in junctional nevi and metastatic melanoma but decreases with dermal content in benign lesions. p16 demonstrates strong, diffuse expression in nevi but is frequently lost in melanoma, although some cases can retain partial expression. Beta‐catenin is present in deep penetrating nevi and may be seen in primary melanomas but may diminish with disease progression. PRAME shows a diffuse nuclear expression in most cases of melanoma (> 75%), while benign nevi exhibit weak or absent staining. Ki‐67 is typically low (< 5%) and confined to the superficial dermis in benign nevi but exceeds 5% and shows a diffuse expression in melanoma. The combined use of these markers enhances diagnostic accuracy, particularly in distinguishing benign nevi from melanoma (Koh et al. 2018; Milman et al. 2020; Saleem et al. 2022).

The development of nevi is a multifactorial and heterogeneous biological process, with the molecular pathways underlying melanocytic neoplasms being diverse and only partially understood (Roh et al. 2015). It is currently established that melanocytic nevi frequently harbor the BRAF^V600E^ mutation, or less commonly, NRAS or HRAS mutations. Once activated, BRAF triggers the mitogen‐activated protein kinase (MAPK) signaling cascade, leading to cell cycle progression, transcriptional upregulation, and cellular differentiation that drive the initial hyperproliferation that results in the formation of the nevus (Meleti et al. 2007; Takata and Saida 2006). The expression of MAPK‐activating mutations in nevi induces oncogene‐induced senescence, thereby limiting cell proliferation and preventing benign nevus cells from invading surrounding keratinocytes (Vredeveld et al. 2012).

While epidemiological studies indicate sun exposure plays a significant role in the development of acquired melanocytic nevi (Gallagher and McLean 1995), the relationship between sun exposure and BRAF^V600E^ mutation remains unclear, as these mutations lack the typical UVB‐induced signatures (Takata and Saida 2006). Given this unclear link between sun exposure and nevi, we investigated whether sun exposure influences the expression of two melanogenesis‐related markers, TRP2 and MiTF, by comparing nevi from sun‐protected regions (intraoral) with those from sun‐exposed areas (vermilion of the lip).

The protein encoded by the *microphthalmia (mi)* gene, known as MiTF, is a transcription factor and a key nuclear component of the signal transduction pathway essential for the development and survival of melanocytes, as the MiTF‐deficient mice lack viable melanocytes (King et al. 2001; Miettinen et al. 2001). UVB exposure is known to upregulate SOX9, a protein that promotes melanocyte differentiation, which in turn activates the MiTF promoter, leading to the expression of TRP2 and tyrosinase (TYR), ultimately enhancing melanin production (D'Mello et al. 2016). Lu et al. (2010) reported higher MiTF expression in sun‐damaged skin with solar elastosis from the extremities, trunk, and head and neck (Lu et al. 2010), whereas King et al. (2001) observed MiTF immunoreactivity in all 62 skin melanocytic nevi (King et al. 2001). In contrast, our study found MiTF upregulated in nevi from both the oral and vermilion lip, suggesting UVB‐induced activation of MiTF does not appear to directly influence the differential development of these lesions from sun‐exposed and sun‐protected areas. This finding challenges the hypothesis that UV radiation plays a primary role in driving MiTF‐associated molecular alterations in OMNs and suggests that alternative regulatory mechanisms—such as intrinsic genetic or epigenetic factors—may contribute to its expression profile in these lesions.

MiTF is recognized as a sensitive and relatively specific marker for melanocytes and melanoma (King et al. 2001). Interestingly, MiTF regulates melanocyte development and functions as both a melanoma oncogene and tumor suppressor, depending on its expression level. While intermediate levels promote melanoma proliferation, high levels inhibit tumor growth and stimulate differentiation, and low levels lead to cell‐cycle arrest and apoptosis. MiTF's role is context‐dependent, influencing melanoma progression and survival (Lu et al. 2010). Previous studies have consistently demonstrated MiTF immunostaining in all benign cutaneous nevi, although Spitz and neurotized nevi show reduced staining intensity. Except for desmoplastic melanomas, all primary and metastatic cutaneous melanomas were also immunopositive, regardless of cell type (Granter et al. 2001; King et al. 2001; Miettinen et al. 2001). Notably, MiTF staining was positive in a few melanomas that failed to stain for either HMB‐45, S‐100, TYR, or Melan‐A which are the most commonly used melanoma markers (King et al. 1999; Miettinen et al. 2001). While MiTF does not distinguish between benign and malignant melanocytic lesions, it remains a highly sensitive and specific histopathological marker for epithelioid melanoma in invasive neoplasms of unknown origin (King et al. 2001, 1999). However, in melanomas with spindle cell morphology, MiTF is less reliable, as it can be expressed in non‐melanocytic spindle cell tumors, including dermatofibromas, schwannomas, leiomyomas, and leiomyosarcomas. However, when diffuse MiTF staining is observed and evaluated in conjunction with clinical, histological, and other immunohistochemical findings, it can be diagnostically useful in select cases of desmoplastic melanoma (Granter et al. 2001).

TRP2 is a melanosomal enzyme with dopachrome tautomerase activity and is a member of the tyrosinase‐related protein family, which also includes TYR and tyrosinase‐related protein‐1 (TRP1) (Lu et al. 2010). The DCT gene, also known as TRP2, encodes an enzyme essential for the eumelanin biosynthesis, catalyzing the conversion of dopachrome to 5,6‐dihydroxyindole‐2‐carboxylic acid (DHICA) (Ainger et al. 2017). A study utilizing IHC and RT in situ PCR demonstrated a higher expression of TRP2 in melanocytic nevi as well as in primary and metastatic melanoma cells (Itakura et al. 2008). In addition to its role in melanogenesis, TRP2 is involved in the cellular response to apoptotic stimuli and oxidative stress, and serves as one of the earliest markers of melanocyte development (Ainger et al. 2017). Both TRP1 and TRP2 also suppress tyrosinase‐mediated cell death of melanocytes and melanoma cells. Even in advanced‐stage, amelanotic melanomas with severely impaired melanin synthesis, pigment‐related proteins, including TRP1 and TRP2, are variably expressed (Itakura et al. 2008). In our study, RT‐qPCR and IHC produced comparable results in detecting TRP2 expression at both oral and in the vermilion lip site. These findings underscore the consistent expression of TRP2 across different anatomical sites, supporting its role in melanocyte function and the pathophysiology of melanocytic lesions. However, the comparable TRP2 expression in nevi from both sun‐exposed and sun‐protected areas suggests that its regulation is independent of UVB radiation, further supporting the hypothesis that additional intrinsic factors—such as genetic predisposition or epigenetic modifications—may drive its expression in OMNs, as for MiTF.

This study has some limitations. First, the sample size is relatively small, which may limit the generalizability of the findings; however, this is reflective of the rarity of these lesions. Moreover, we needed to select only the cases containing enough lesion tissue to perform all the proposed analyses. In this regard, future studies might benefit from expanding the sample size or including samples from multiple centers to improve the generalizability of the results. Second, while TRP2 and MiTF were analyzed using both immunohistochemistry and RT‐qPCR, other melanogenesis‐related markers and potential UVB‐responsive genes were not assessed, restricting the conclusions about UV exposure's impact on OMNs. Additional factors beyond TRP2 and MiTF may also influence the observed characteristics. Third, the cross‐sectional design of this study limits conclusions about causation or progression of these lesions. Lastly, the use of archived FFPE samples, although valuable for molecular analysis, presents inherent challenges such as potential RNA/DNA degradation, which may affect the reliability of molecular results. However, to mitigate this issue, only samples with high quality and quantity of RNA were included in the study.

In conclusion, OMNs are rare neoplasms, and distinct histopathological differences are observed between those located inside the oral cavity and those located in the vermilion lip. Nevi on the vermilion lip exhibit greater pleomorphism, a UVB‐induced stromal reaction (solar elastosis), and are characterized by symmetrical cell arrangement in the theques with floret cells. In contrast, intraoral nevi tend to be asymmetrical, lack lateral circumscription, and do not form abundant theques. However, they show no signs of mitotic activity, pleomorphism, or inflammatory response. Additionally, TRP2 and MiTF are upregulated in nevi from both the intraoral and vermilion lip. These findings suggest that while there are notable histopathological differences between OMNs in sun‐exposed and sun‐protected areas, the molecular and protein expression of key melanogenesis markers TRP2 and MiTF remains consistent in the oral cavity and vermilion lip nevi. Therefore, it is probable that factors other than UVB exposure may be driving these molecular features or UVB radiation influences other melanocytic genes not evaluated here.

## Author Contributions

**Thalita Soares Tavares:** conceptualization, investigation, writing – original draft, methodology, formal analysis, data curation. **Adriana Aparecida Silva da Costa:** investigation, methodology. **Marina Gonçalves Diniz:** investigation, methodology, validation, writing – review and editing, formal analysis, data curation. **Daniela Pereira Meirelles:** investigation, methodology. **Sandra Beatriz Chaves Tarquínio:** methodology. **Ana Carolina Uchoa Vasconcelos:** methodology. **Aline Cristina Batista Rodrigues Johann:** methodology. **Ricardo Santiago Gomez:** investigation, methodology, resources. **Maria Cássia Ferreira de Aguiar:** investigation, methodology, writing – review and editing. **Patrícia Carlos Caldeira:** conceptualization, investigation, funding acquisition, writing – review and editing, validation, formal analysis, project administration, supervision, resources.

## Ethics Statement

This study was performed in accordance with the Declaration of Helsinki and was approved by the Committee of Ethics in Research of *Universidade Federal de Minas Gerais* (certificate number 57392522.6.0000.5149).

## Consent

All the authors gave their consent for publication.

## Conflicts of Interest

The authors declare no conflicts of interest.

## Supporting information


Table S1.

Table S2.

## Data Availability

The data that support the findings of this study are available on request from the corresponding author. The data are not publicly available due to privacy or ethical restrictions.
