# Supplementary material for: Characterization of Oral Melanocytic Nevi in Sun‐Exposed and Sun‐Protected Regions
Source: Oral Dis. 2025 Jun 12;31(10):2906–13. doi: 10.1111/odi.15404 (PMC12721702; doi:10.1111/odi.15404)
Supplement: Supplementary file 1 — Table S1. [file ODI-31-2906-s002.docx]

**Table 1 -** Clinical and Demographic Characteristics of Intraoral and Vermilion Lip Nevi

|  | **Intraoral (n= 14)** | | | | **The vermilion of the lip (n= 20)** | | | |
| --- | --- | --- | --- | --- | --- | --- | --- | --- |
|  | **Compound (n= 3)** | **Intramucosal (n= 5)** | **Blue (n= 6)** | **All (n= 14)** | **Compound (n= 6)** | **Intramucosal (n= 13)** | **Blue (n= 1)** | **All (n= 20)** |
| **Sex** |  |  |  |  |  |  |  |  |
| Female | 3 | 3 | 4 | 10 (71.4%) | 2 | 11 | 1 | 14 (70.0%) |
| Male | 0 | 2 | 2 | 4 (28.6%) | 4 | 2 | 0 | 6 (30.0%) |
| **Age** |  |  |  |  |  |  |  |  |
| 0-10 | 2 | 0 | 0 | 2 (14.3%) | 1 | 0 | 0 | 1 (5.0%) |
| 11-20 | 1 | 1 | 1 | 3 (21.4%) | 2 | 2 | 0 | 4 (20.0%) |
| 21-30 | 0 | 1 | 1 | 2 (14.3%) | 0 | 2 | 0 | 2 (10.0%) |
| 31-40 | 0 | 1 | 2 | 3 (21.4%) | 0 | 3 | 1 | 4 (20.0%) |
| 41-50 | 0 | 1 | 2 | 3 (21.4%) | 2 | 3 | 0 | 5 (25.0%) |
| 51-60 | 0 | 0 | 0 | 0 (0.0%) | 1 | 1 | 0 | 2 (10.0%) |
| 61-70 | 0 | 1 | 0 | 1 (7.1%) | 0 | 1 | 0 | 1 (5.0%) |
| Missing | 0 | 0 | 0 | 0 (0.0%) | 0 | 1 | 0 | 1 (5.0%) |
| **Race** |  |  |  |  |  |  |  |  |
| White | 1 | 2 | 1 | 4 (28.6%) | 1 | 13 | 1 | 15 (75.0%) |
| Brown | 2 | 2 | 2 | 6 (42.9%) | 3 | 0 | 0 | 3 (15.0%) |
| Black | 0 | 1 | 2 | 3 (21.4%) | 1 | 0 | 0 | 1 (5.0%) |
| Missing | 0 | 0 | 1 | 1 (7.1%) | 1 | 0 | 0 | 1 (5.0%) |
| **Location** | Alveolar ridge (n= 1), Gingiva (n=1), Retromolar trigon (n=1) | Buccal mucosa (n= 2), Soft palate (n= 1), Gingiva (n= 1), Oral vestibule (n= 1) | Hard palate (n=5), Palate (n= 1) | Hard palate (n=5, 35.7%), Gingiva (n= 2, 14.3%), Buccal mucosa (n= 2, 14.3%), Soft palate (n= 1, 7.1%), Palate (n= 1, 7.1%), Oral vestibule (n= 1, 7.1%), Retromolar trigon (n=1, 7.1%), Alveolar ridge (n= 1, 7.1%) | Lower lip (n= 5), Upper lip (n=1) | Lower lip (n= 6), Upper lip (n= 6), Lip (n= 1) | Lower lip (n= 1) | Lower lip (n= 12, 60.0%), Upper lip (n= 7, 35.0%), Lip (n= 1, 5.0%) |
| **Clinical appearance** |  |  |  |  |  |  |  |  |
| Macule | 2 | 3 | 4 | 9 (64.3%) | 0 | 1 | 0 | 1 (5.0%) |
| Papule | 0 | 1 | 0 | 1 (7.1%) | 1 | 3 | 0 | 4 (20.0%) |
| Nodule | 0 | 0 | 0 | 0 (0.0%) | 1 | 0 | 1 | 2 (10.0%) |
| Missing | 1 | 1 | 2 | 4 (28.6%) | 4 | 9 | 0 | 13 (65.0%) |
| **Color** |  |  |  |  |  |  |  |  |
| Black | 2 | 2 | 4 | 8 (57.1%) | 2 | 2 | 0 | 4 (20.0%) |
| Brown | 1 | 0 | 0 | 1 (7.1%) | 1 | 3 | 0 | 4 (20.0%) |
| Purple | 0 | 1 | 2 | 3 (21.4%) | 0 | 0 | 0 | 0 (0.0%) |
| Red | 0 | 2 | 0 | 2 (14.3%) | 0 | 0 | 0 | 0 (0.0%) |
| Pink | 0 | 0 | 0 | 0 (0.0%) | 1 | 3 | 1 | 5 (25.0%) |
| White | 0 | 0 | 0 | 0 (0.0%) | 1 | 0 | 0 | 1 (5.0%) |
| Missing | 0 | 0 | 0 | 0 (0.0%) | 1 | 5 | 0 | 6 (30.0%) |
| **Size (mm)** |  |  |  |  |  |  |  |  |
| 0-5 | 0 | 2 | 6 | 8 (57.1%) | 3 | 7 | 0 | 10 (50.0%) |
| 6-10 | 2 | 2 | 0 | 4 (28.6%) | 0 | 0 | 1 | 1 (5.0%) |
| 11-15 | - | - | - |  | - | - | - | 0 (0.0%) |
| 16-20 | 1 | 0 | 0 | 1 (7.1%) | 0 | 1 | 0 | 1 (5.0%) |
| Missing | 0 | 1 | 0 | 1 (7.1%) | 3 | 5 | 0 | 8 (40.0%) |
