# Supplementary material for: Characterization of Oral Melanocytic Nevi in Sun‐Exposed and Sun‐Protected Regions
Source: Oral Dis. 2025 Jun 12;31(10):2906–13. doi: 10.1111/odi.15404 (PMC12721702; doi:10.1111/odi.15404)
Supplement: Supplementary file 2 — Table S2. [file ODI-31-2906-s001.docx]

**Table 2 –** Histological Features of Intraoral and Vermilion Lip Nevi

|  | | **Intraoral (n= 14)** | | | | **The vermilion of the lip (n= 20)** | | | |
| --- | --- | --- | --- | --- | --- | --- | --- | --- | --- |
|  | | **Compound (n= 3)** | **Intramucosal (n= 5)** | **Blue (n= 6)** | **All (n= 14)** | **Compound (n= 6)** | **Intramucosal (n= 13)** | **Blue (n= 1)** | **All (n= 20)** |
| **Cell morphology** | |  |  |  |  |  |  |  |  |
| Round | 2 | 5 | 0 | 7 (50.0%) | 5 | 13 | 0 | 18 (90.0%) |  |
| Fusiform | 0 | 0 | 6 | 6 (42.9%) | 0 | 0 | 1 | 1 (5.0%) |  |
| Round/Fusiform | 1 | 0 | 0 | 1 (7.1%) | 0 | 0 | 0 | - |  |
| Fusiform/Round | 0 | 0 | 0 | - | 1 | 0 | 0 | 1 (5.0%) |  |
| **Side circumscription** | |  |  |  |  |  |  |  |  |
| Present | | 2 | 2 | 1 | 5 (35.7%) | 2 | 7 | 0 | 9 (45.0%) |
| Absent | | 1 | 3 | 5 | 9 (64.3%) | 4 | 6 | 1 | 11 (55.0%) |
| **Symmetry** | |  |  |  |  |  |  |  |  |
| Present | | 2 | 2 | 2 | 6 (42.9%) | 4 | 12 | 1 | 17 (85.0%) |
| Absent | | 1 | 3 | 4 | 8 (57.1%) | 2 | 1 | 0 | 3 (15.0%) |
| **Inflammation** | |  |  |  |  |  |  |  |  |
| Present | | 0 | 0 | 1 | 1 (7.1%) | 1 | 4 | 0 | 5 (25.0%) |
| Absent | | 3 | 5 | 5 | 13 (92.9%) | 5 | 9 | 1 | 15 (75.0%) |
| **Fibrosis** | |  |  |  |  |  |  |  |  |
| Present | | 1 | 2 | 2 | 5 (35.7%) | 2 | 10 | 1 | 13 (65.0%) |
| Absent | | 2 | 3 | 4 | 9 (64.3%) | 4 | 3 | 0 | 7 (35.0%) |
| **Solar elastosis** | |  |  |  |  |  |  |  |  |
| Present | | - | - | - | - | 1 | 5 | 0 | 6 (30.0%) |
| Absent | | - | - | - | - | 5 | 8 | 1 | 14 (70.0%) |
| Not applicable | | 3 | 5 | 6 | 14 (100.0%) | - | - | - | - |
| **Theques formation** | |  |  |  |  |  |  |  |  |
| Present | | 2 | 1 | 0 | 3 (21.4%) | 5 | 11 | 0 | 16 (80.0%) |
| Absent | | 1 | 4 | 6 | 11 (78.6%) | 1 | 2 | 1 | 4 (20.0%) |
| **Floret cell** | |  |  |  |  |  |  |  |  |
| Present | | 0 | 1 | 0 | 1 (7.1%) | 4 | 9 | 0 | 13 (65.0%) |
| Absent | | 3 | 4 | 6 | 13 (92.9%) | 2 | 4 | 1 | 7 (35.0%) |
| **Rete ridge** | |  |  |  |  |  |  |  |  |
| Without alteration | | 1 | 5 | 6 | 12 (85.7%) | 6 | 11 | 1 | 18 (90.0%) |
| Elongated | | 0 | 0 | 0 | 0 (0.0%) | 0 | 1 | 0 | 1 (5.0%) |
| Elongated + bridge | | 2 | 0 | 0 | 2 (14.3%) | 0 | 1 | 0 | 1 (5.0%) |
| **Pleomorphism** | |  |  |  |  |  |  |  |  |
| Absent | | 2 | 3 | 4 | 9 (64.3%) | 1 | 2 | 1 | 4 (20.0%) |
| Mild | | 0 | 1 | 2 | 3 (21.4%) | 2 | 6 | 0 | 8 (40.0%) |
| Moderate | | 1 | 0 | 0 | 1 (7.1%) | 2 | 5 | 0 | 7 (35.0%) |
| Intense | | 0 | 1 | 0 | 1 (7.1%) | 1 | 0 | 0 | 1 (5.0%) |
| **Mitosis** | |  |  |  |  |  |  |  |  |
| Present | | 0 | 0 | 0 | 0 (0.0%) | 1 | 0 | 0 | 1 (5.0%) |
| Absent | | 3 | 5 | 6 | 14 (100.0%) | 5 | 13 | 1 | 19 (95.0%) |
| **Melanin** | |  |  |  |  |  |  |  |  |
| Absent | | 0 | 0 | 0 | 0 (0.0%) | 2 | 2 | 0 | 4 (20.0%) |
| Mild | | 2 | 3 | 2 | 7 (50.0%) | 2 | 9 | 0 | 11 (55.0%) |
| Moderate | | 1 | 1 | 3 | 5 (35.7%) | 1 | 2 | 1 | 4 (20.0%) |
| Intense | | 0 | 1 | 1 | 2 (14.3%) | 1 | 0 | 0 | 1 (5.0%) |
